# Acute effects of ingesting a commercial thermogenic drink on changes in energy expenditure and markers of lipolysis

**DOI:** 10.1186/1550-2783-5-6

**Published:** 2008-02-20

**Authors:** Vincent J Dalbo, Michael D Roberts, Jeffrey R Stout, Chad M Kerksick

**Affiliations:** 1 Department of Health and Exercise Science, University of Oklahoma, Norman, OK, USA

## Abstract

**Background:**

To determine the acute effects of ingesting a thermogenic drink (Celsius, Delray Beach, FL) (TD) on changes in metabolism and lipolysis.

**Methods:**

Healthy college-aged male (23.2 ± 4.0 y, 177.2 ± 6.1 cm, 81.7 ± 11.3 kg, 22.8 ± 7.3 % fat; n = 30) and female (23.4 ± 3.1 y, 165.6 ± 8.7 cm, 62.1 ± 9.9 kg, 28.3 ± 7.4 % fat; n = 30) participants were matched according to height and weight to consume 336 ml of the TD or a non-caloric, non-caffeinated placebo (PLA). After a 12 h fast, participants reported for pre-consumption measures of height, weight, heart rate, blood pressure, resting energy expenditure (REE), respiratory exchange ratio (RER), glycerol and free-fatty acid (FFA) concentrations. REE and RER were determined at 60, 120, and 180 min post-consumption. Serum glycerol and FFA concentrations were determined at 30, 60, 120 and 180 min post-consumption.

**Results:**

When compared to PLA, TD significantly increased REE at 60, 120 and 180 min (p < 0.05). FFA concentrations were significantly greater in TD compared to PLA at 30, 60, 120 and 180 min post-consumption (p < 0.05). No between-group differences were found in RER.

**Conclusion:**

Acute TD ingestion significantly increased REE, FFA and glycerol appearance. If sustained, these changes may help to promote weight loss and improve body composition; however, these findings are currently unknown as are the general safety and efficacy of prolonged consumption.

## Background

The prevalence of individuals who are classified as overweight and obese is a primary health concern due to their relationship to various cardiovascular diseases [1,2] and associated comorbidities [1]. Recent epidemiological data suggests that 31% of the United States (U.S.) population is classified as obese, according to body mass index (BMI) standards, while 65% is classified as overweight [3]. Medical expenses for obesity treatment and its related comorbities in 1998 accounted for 9.1% of total medical expenditures and was reported to be $78.5 billion, which would exceed $100 billion in today's economy [4]. In addition to reducing medical costs, weight loss can reduce risk for cardiovascular disease, improve quality of life and ease the economic burden due to missed work and diminished work productivity [2,5,6]. Weight loss can be achieved by maintaining a proper diet and or starting an exercise regime; however, adherence to conventional weight-loss programs has been poor [3]. As a result the use of nutritional supplements to aid weight loss has increased in popularity, with consumers spending an estimated $37.1 billion per year [7].

Many consumers are turning to thermogenic drinks as a means to increase calorie and fat burning, energy levels and weight loss. While the number of TD has increased in recent years, they typically contain a mixture of caffeine, catechins, various herbal extracts, amino acids, neurostimulants and vitamins. Ingredients in these products have been shown to increase metabolism [8-13], decrease body fat [14,15] and increase markers of lipolysis [14,16-19]. Furthermore, recent work has affirmed that a TD containing caffeine, citrus aurantium, garcinia, cambogia and chromium polynicotinate to increase calorie expenditure [13,20]. However, limited research has been reported on the impact of ingesting a TD in either an acute or prolonged fashion on energy expenditure and circulating markers of lipolysis. Furthermore, the potential for a TD to promote acute increases in fat metabolism appear promising as a recently published study reported a TD containing tyrosine, capsaicin, catechins and caffeine increased resting metabolism 4 hours post consumption compared to a placebo drink [21]. Therefore, the purpose of this study was to examine the acute effects of ingesting a commercially available TD (Celsius, Celsius, Inc., Delray Beach, FL) for changes in resting energy expenditure (REE) and serum markers of lipolysis. It was hypothesized that acute TD ingestion would increase resting energy expenditure and serum markers of lipolysis when compared to a placebo (PLA).

## Methods

### Participants

Healthy college-aged males (n = 30) and females (n = 30) participated in this study (Table 1). All testing was conducted after the participant signed the IRB-approved informed consent and completed comprehensive medical history questionnaires. Participants were excluded if they: 1) had a history of hypertension, metabolic, hepatorenal, musculoskeletal, autoimmune or neurological disease; 2) were currently taking thyroid, antihyperlipidemic, hypoglycemic, anti-hypertensive or androgenic medications; or 3) had consumed nutritional supplements that may affect metabolism [i.e., over 100 mg·d^-1 ^of caffeine, ephedrine alkaloids, guggulsterones, etc.] and/or muscle mass [i.e. creatine, protein/amino acids, androstenedione, dihydroepiandrosterone (DHEA), etc.] within three months of starting the study.

**Table 1:** Subject demographics

Variable	TD (n = 30)	PLA (n = 30)	Significance
Age (y)	23.3 ± 0.6	23.3 ± 0.7	1.00
Height (cm)	172.1 ± 1.8	170.7 ± 1.6	0.56
Body mass (kg)	72.6 ± 2.6	71.2 ± 2.7	0.71
Systolic BP (mm Hg)	124 ± 2	121 ± 3	0.51
Diastolic BP (mm Hg)	78 ± 2	77 ± 2	0.49
Heart Rate (beats·min^-1^)	71 ± 3	72 ± 2	0.77

TD, Thermogenic drink; PLA, placebo. Values are expressed as means ± SE.

### Experimental design

Participants reported to the laboratory for a single testing session following completion of a 2-day food diary, a 12 h overnight fast and 24 h avoidance of exercise. Upon arrival, participants were matched into clusters according to age and fat free mass and distributed evenly among both groups. Participants were then assigned in a single-blind fashion to ingest in a daily fashion isovolumetric amounts (336 ml) of either the thermogenic drink (TD) or placebo (PLA). The PLA drink was a commercially available carbonated, non-caloric, non-caffeinated beverage. Measures of body mass, height, blood pressure, heart rate, percent body fat, REE, glycerol and free fatty acids were obtained.

### Anthropometric measures

After voiding, participants changed into minimal clothing and were barefoot for measurement of body mass and height on a calibrated scale and standiometer (Detecto, Webb City, MO). Body mass and height were measured to the nearest 0.05 kg and 0.5 cm, respectively. For measurement of height participants were instructed to stand erect, inhale deeply, point toes up and look straight ahead. After resting for 5 min, heart rate and blood pressure were obtained according to standard procedures using an automated blood pressure monitor (Omron HealthCare, Inc., HEM-757, Vernon Hills, IL). Omron HealthCare, Inc. reports the HEM-757 to have a standard deviation of ± 3 mmHg for blood pressure and to be within ± 5% of pulse rate.

### Body composition

Measures of percent body fat were obtained using a BOD POD^® ^(Life Measurement, Inc., Concord, CA). Prior to each test the BOD POD^® ^was calibrated according to the manufacturer's instructions. Participants then changed into a tight fitting bathing suit and swim cap before sitting in the BOD POD^®^. The BOD POD^® ^was then sealed while the subject sat motionless and breathed normally for 20 sec while body volume was measured. Thoracic gas volume correction was performed according to standard procedures. Our lab has previously demonstrated total error of measurement values of 0.66% body fat.

### Resting energy expenditure and respiratory exchange ratio

Resting energy expenditure was determined to assess changes in whole body energy expenditure after drink ingestion. Participants were led to a quiet, dark, thermally controlled room and were instructed to lie in a supine position. A clear, hard plastic breathing hood with a clear plastic drape was attached to a metabolic cart (ParvoMedics TrueOne^® ^2400, Sandy, UT) and placed over the participants head and upper torso. Mean oxygen uptake (VO_2_) and carbon dioxide output (VCO_2_) were measured for each breath and averaged over 15 sec intervals. REE and RER were measured for 20 minutes and reported data represent a 5 min window at the end of the collection period in which a criterion variable (VO_2_) deviated by less than 5%. Test-retest correlations (*r*) using this device were in the range of 0.550 – 0.747 with a mean intra-class coefficient of 0.893, p < 0.001.

### Venous blood sampling

Venous blood samples were taken from an antecubital vein using standard phlebotomy techniques with a 22-gauge needle and a 6 mL serum separation vacutainer tube (BD Vacutainer^®^, Franklin Lakes, NJ). Each vacutainer was inverted several times and immediately centrifuged at 3,500 rpm for 15 min. The resulting supernatant was then aliquoted into two microcentrifuge tubes and stored at -20°C for subsequent analysis.

### Glycerol and free fatty acid analyses

To assess acute changes in serum levels of lipolysis, serum concentrations of glycerol and FFAs were determined in duplicate. Glycerol was assessed using a calibrated commercial oxidase enzyme reaction analyzer and reagents (Analox GM7, Analox Instruments, London, England). FFAs were assessed using a commercial colorimetric assay (Roche, Penzeberg, Germany) and read with a spectrophotometer (SmartSpec™Plus, Bio-Rad, Hercules, CA) at a wavelength of 546 nm. Assay precision (coefficient of variation (C_*V*_)) and accuracy (percent of un-recovery) were calculated using control samples for glycerol and FFAs by 8 replicate determinations. The reported C_*V *_and percent of un-recovery for glycerol (240 μM control serum: 2.5%, 1.2%) and free fatty acid control serums (0.35 mM control serum: 8.9%, 3.6%) were found to be within acceptable ranges [22,23].

### Statistical analysis

Separate two-way (group × time) repeated measures ANOVAs on raw data were first used to determine differences between genders, testing sessions, and interactions. Delta values (testing point – baseline) were calculated for relative REE, glycerol content, free fatty acid content and are displayed in all figures. When necessary, additional one-way ANOVAs were used to further evaluate any significant group × time interactions. Baseline demographic, body composition and dietary intake data were analyzed using independent t-tests. When the sphericity assumption was not met, the conservative Hunyh-Feldt Epsilon correction factor was used to evaluate observed within-group F ratios. A probability level of ≤ 0.05 was to determine significance.

## Results

### Subject demographics and nutritional data

No baseline significant differences were found between age, height, weight, body composition (Table 1). Additionally, no statistical differences between groups were found for caloric intake (TD: 25.78 ± 8.49 vs. PLA: 29.15 ± 13.29 kcal·kg·d^-1^; p = 0.21), protein (TD: 1.16 ± 0.44 vs. PLA: 1.27 ± 0.78 g·kg·d^-1^; p = 0.50), carbohydrate (TD: 3.27 ± 1.16 vs. PLA: 3.73 ± 1.67 g·kg·d^-1^; p = 0.23) or fat (TD: 0.91 ± 0.39 vs. PLA: 1.07 ± 0.60 g·kg·d^-1^; p = 0.23) intake.

### Acute changes in REE and RER

**Figure 1:**
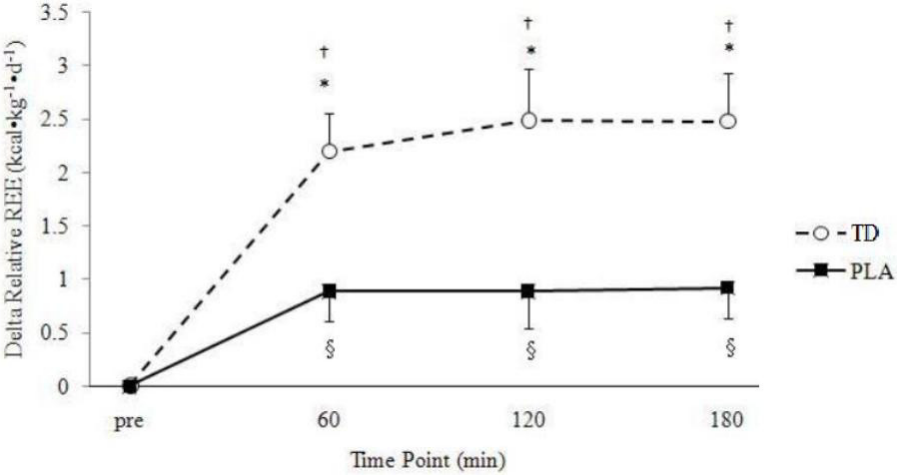
**Delta changes in relative REE (kcal·kg^-1^·d^-1^).** Data is expressed as means ± SE. † TD greater than baseline (p < 0.05). § PLA greater than baseline (p < 0.05). * TD > PLA (p < 0.05). TD, thermogenic drink (n = 30); PLA, placebo (n = 30).

A significant group × time interaction (p = 0.004) was found for relative REE (Figure 1). No main effect for group was found while additional one-way ANOVAs revealed significant between group differences for delta change in relative REE at 60 (p = 0.005), 120 (p = 0.009) and 180 (p = 0.005) min. Additionally, a significant group × time interaction (p = 0.004) was found for the raw REE (kcals·d^-1^) data in addition to a main effect for time (p < 0.001; Table 2). However, no main effect for group was found (p = 0.089). No significant group × time interaction was found for RER; however, a significant main effect for time (p < 0.001) was found (Table 2).

**Table 2:** Energy expenditure data

Variable	Time	PLA (n = 30)	TD (n = 30)	Significance	
RER	Pre	0.83 ± 0.08	0.81 ± 0.08	Group	0.870
	60 min	0.82 ± 0.09	0.81 ± 0.10	Time	< 0.001*
	120 min	0.79 ± 0.07	0.80 ± 0.07	G × T	< 0.860
	180 min	0.78 ± 0.07	0.78 ± 0.11		
					
REE (kcals·d^-1^)	Pre	1581 ± 337	1636 ± 307§	Group	± 0.090
	60 min	1641 ± 325	1782 ± 278§	Time	< 0.001*
	120 min	1632 ± 295	1801 ± 283§	G × T	± 0.004§
	180 min	1639 ± 325	1802 ± 296§		

TD, thermogenic drink; PLA, placebo; Data are means ± SE.

* Significant main effect for time (p < 0.05).

§ TD > PLA (p < 0.05).

### Acute glycerol and FFA levels

**Figure 2:**
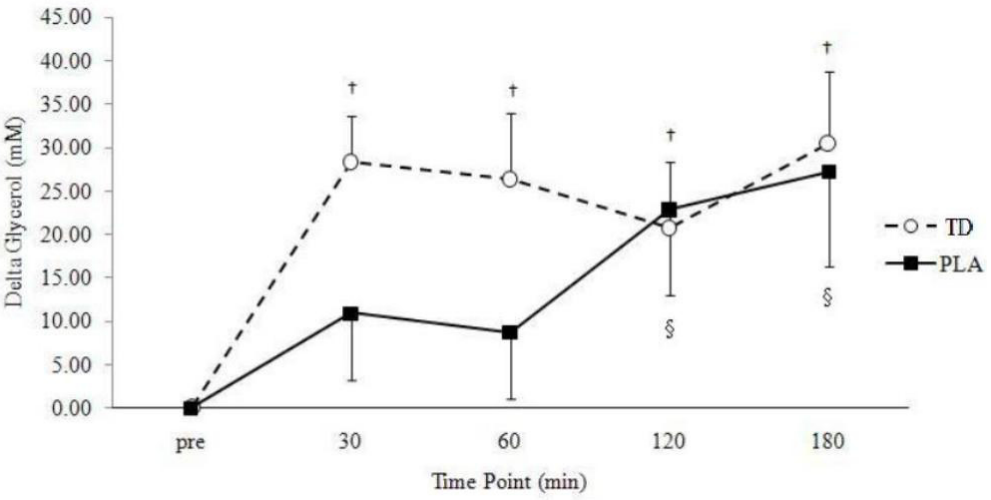
**Delta changes in serum glycerol concentrations (mM).** Data is expressed as means ± SE. † TD greater than baseline (p < 0.05). § PLA greater than baseline (p < 0.05). TD, thermogenic drink (n = 30); PLA, placebo (n = 30).

**Figure 3:**
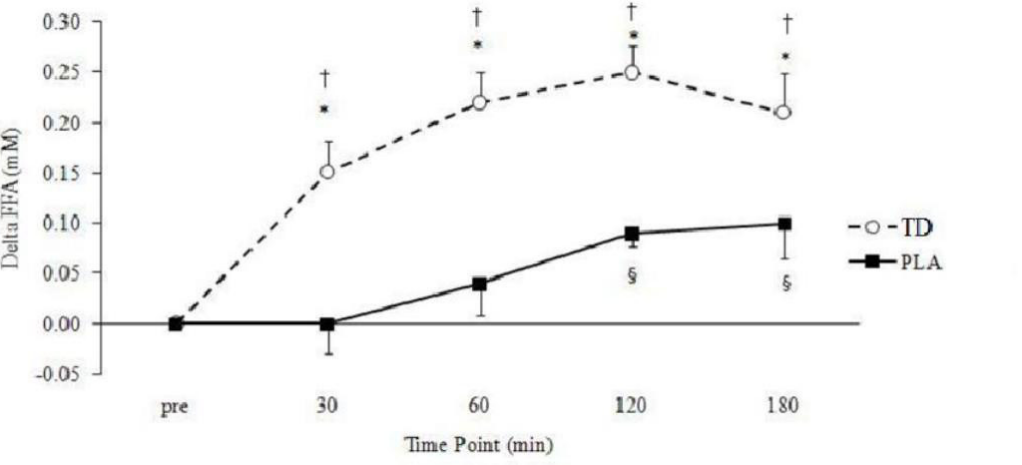
**Delta changes in serum free fatty acid concentrations (mM).** Data is expressed as means ± SE. † Significantly greater than baseline (p < 0.05). * TD > PLA (p < 0.05). § PLA greater than baseline (p < 0.05). TD, thermogenic drink (n = 30); PLA, placebo (n = 30).

A significant group × time interaction (p = 0.001) was found for FFA (Figure 2) while no such changes was found in serum glycerol levels (p = 0.18; Figure 3). Significant main effects for time were found for changes in FFA (p < 0.001) and glycerol (p < 0.001), revealing similar increases in both groups across time. One-way ANOVAs revealed significantly higher levels of circulating FFA at 30 (p = 0.001), 60 (p < 0.001), 120 (p < 0.001) and 180 min (p = 0.03) following CEL ingestion when compared to PLA. Additional one-way ANOVAs revealed a trend toward significantly higher levels of circulating glycerol at 30 min (p = 0.054) following CEL ingestion compared to PLA with no other reported changes.

## Discussion

The purpose of this study examined the acute effects of ingesting a commercially available thermogenic drink (TD) on energy expenditure and lipolysis independent of diet and/or exercise modifications. Upon considering previous animal and human research, we hypothesized that energy expenditure, fat oxidation and serum levels of lipolysis markers would acutely increase after ingestion. Of the active ingredients, caffeine and epigallocatechin gallate (EGCG, an abundant catechin in green tea) [24] are the most common and have been found to increase energy expenditure [8,10,11,25-29] and increase markers of lipolysis [8-11]. In this regard, caffeine increases energy metabolism through stimulation of β-adrenergic receptors and adenyl cyclase resulting in activation of cyclic AMP (cAMP), causing subsequent increases in circulating epinephrine [30,31] and free fatty acids [32]. Caffeine may also enhance lipolysis by inhibiting nucleotide phosphodiesterase resulting in increased tissue concentrations of cAMP and activation of hormone-sensitive lipase, which provides further explanation for the increased circulating levels of free fatty acids seen in the present study [12]. In this regard, caffeine doses of 6 mg·kg^-1 ^in individuals with impaired epinephrine responses have been shown to increase FFA and glycerol concentration independent of a significant change in epinephrine [33]. An additional bioactive ingredient of importance is the green tea catechin, EGCG. Recent human and animal investigations have demonstrated various positive health effects of EGCG administration. For example, Boschmann gave 300 mg·d^-1 ^of EGCG alone to six obese men and measured energy expenditure and respiratory quotient changes [34]. While overall energy expenditure did not change, the respiratory quotient numbers did lower in response to EGCG administration leading the authors to conclude that EGCG alone may be beneficial to increase fat oxidation, although no biochemical measures of fat oxidation were measured. Furthermore, Hill and colleagues reported an improved heart rate and glycemic response in obese, postmenopausal women after 12 weeks of EGCG consumption (150 mg·d^-1^) along with a walking exercise program [35]. In a more mechanistic fashion, recent studies have also suggested EGCG may alter food digestibility [15,36] and downregulate stearoyl-CoA desaturase (SCD1) gene expression [15], which may reduce adiposity, decrease lipid synthesis and increase liver fatty acid oxidation, as was found in SCD1 knockout mice [15,37].

In support of our hypothesis, acute TD consumption significantly increased relative REE at 60, 120 and 180 min post-ingestion when compared to a non-caloric, non-caffeinated placebo. When this data was not normalized to body mass (kcals·d^-1^) and expressed relative to baseline levels, the TD group burned an average of 106 more calories when compared to PLC over the 3 h testing period (data not shown). When expressed as a percent increase from baseline, calorie burning in the TD group was increased by an average of 10.45% whereby calorie burning in the PLC group was only 0.89% (data not shown). In accordance with these findings, Rudelle et al. (2007) reported a 4.6% increase of energy expenditure 24 h after ingesting a thermogenic drink containing similar ingredients ([38]. Further, single 100 mg doses of caffeine have been shown to increase metabolic rate 3–4%, while a titrated dosing pattern of 6 × 100 mg over a 12 h period may increase energy expenditure 8–12% [10]. Similar changes to these were found in the present study and are likely due to the combination of caffeine and EGCG provided by the energy drink. In a contrasting fashion, substrate oxidation was hypothesized to change following TD ingestion as prior research has found caffeine to positively increase FFA concentrations [8,28,39,40] and fat oxidation [8,9,11,28]. In support of this research, FFA concentrations from the present study were significantly greater following TD consumption compared to PLA at 30, 60, 120 and 180 min post-ingestion (Figure 3). Increased FFA concentrations in the TD group are likely explained by the stimulatory effects of caffeine on circulating epinephrine [41] and subsequent downstream activation of lipolysis by way of the β-adrenergic receptors.

From a biochemical perspective, changes in serum concentrations of glycerol were not as prominent as FFA concentrations. These results, however, are consistent with prior research which has found FFA concentrations to be significantly elevated up to 180 min post-ingestion while glycerol values were only significantly elevated 60 min post-ingestion [37]. Another study found FFA concentrations to be significantly elevated 60 min post-ingestion with a trend toward increased glycerol concentrations 60 min post-ingestion. There were no changes in glycerol release from skeletal muscle in participants consuming caffeine suggesting fat oxidation was not enhanced in skeletal muscle [42] and that a tissue-specific lipolytic response may impact overall serum concentrations. It has been hypothesized that increased glycerol concentrations following caffeine ingestion are most likely a function adenosine-receptor antagonism, resulting in glycerol release from adipose tissue [37].

The results of this study suggest that the proprietary thermogenic blend contained in the TD may be an effective stimulus to promote weight loss independent of modifications in diet or exercise, as TD consumption increased REE, calorie burning and circulating levels of FFA. Due to the acute nature of this study, however, it is not known if the proprietary blend contained in this TD is effective at decreasing body fat as the effects of the ingredients may diminish with chronic use. As a result more prolonged studies should seek to examine the impact of daily TD ingestion with or without diet and exercise changes to determine changes in body mass as well as body composition along with markers of lipolysis and metabolism.

## Conclusion

Acute TD ingestion appears to stimulate significant increases in energy expenditure and circulating levels of FFA while substrate oxidation and circulating levels of glycerol experienced non-significant decreases across time. Additional research needs to further examine the impact of prolonged daily ingestion of TD on energy expenditure, changes in body composition and efficacy of daily ingestion of TD. These studies will provide additional evidence for any role TD may play at promoting weight loss and/or achieving weight maintenance.

## Competing interests

Celsius Holding, Incorporated (Delray Beach, FL) provided funding for this project through a research grant to the University of Oklahoma. All researchers involved independently collected, analyzed, and interpreted the results from this study and have no financial interests concerning the outcome of this investigation.

## Authors' contributions

VJD: primary author, recruitment, data analysis, statistical analysis and manuscript preparation. MDR: manuscript preparation, recruitment, data analysis, statistical analysis. JRS: secondary author who helped obtain grant funds for the project. CMK: principal investigator of the study who obtained grant funds for project, supervised data collection and analysis, supervised statistical analyses, and co-authored paper. All authors have read and approved the final manuscript
